# Supplementary material for: Changes in Serum Levels of Ketone Bodies and Human Chorionic Gonadotropin during Pregnancy in Relation to the Neonatal Body Shape: A Retrospective Analysis
Source: Nutrients. 2022 May 9;14(9):1971. doi: 10.3390/nu14091971 (PMC9099686; doi:10.3390/nu14091971)
Supplement: Supplementary file 1 [file nutrients-14-01971-s001.zip › nutrients-1675626-supplementary.pdf]

Table S1. Human chorionic gonadotropin data of 245 pregnant women.

	Number	Human chorionic gonadotropin
**Blood test, week**		
7	1	164,000
8	68	169,000 (63,700-320,000)
9	47	156,000 (149,300-341,000)
10	48	116,500 (45,700-272,000)
11	45	99,800 (4,640-204,000)
12	26	97,800 (13,800-167,000)
13	9	85,700 (2,620-126,000)
15	1	36,000
23	14	19,600 (3,520-121,000)
24	55	15,000 (564-73,500)
25	53	19,500 (166-216,000)
26	77	14,700 (1,890-92,900)
27	42	19,750 (7,020-63,900)
28	3	11,600 (8,070-37,300)
29	1	78,000
31	1	27,700
34	1	4,040
35	56	18,150 (195-97,400)
36	178	23,050 (32-113,000)
37	8	29,750 (1,780-52,000)
38	1	7,620

Blood data are presented as median (range).
